# Plasma Electrolytic Oxidation (PEO) Process—Processing, Properties, and Applications

**DOI:** 10.3390/nano11061375

**Published:** 2021-05-22

**Authors:** Soumya Sikdar, Pramod V. Menezes, Raven Maccione, Timo Jacob, Pradeep L. Menezes

**Affiliations:** 1 Department of Mechanical Engineering, University of Nevada, Reno, NV 89557, USA; soumyasikdar@nevada.unr.edu (S.S.); rmaccione@nevada.unr.edu (R.M.); 2 Institute of Electrochemistry, Ulm University, Albert-Einstein-Allee 47, 89081 Ulm, Germany; timo.jacob@uni-ulm.de

**Keywords:** plasma electrolytic oxidation, nanocomposite coating, tribology, additives, corrosion

## Abstract

Plasma electrolytic oxidation (PEO) is a novel surface treatment process to produce thick, dense metal oxide coatings, especially on light metals, primarily to improve their wear and corrosion resistance. The coating manufactured from the PEO process is relatively superior to normal anodic oxidation. It is widely employed in the fields of mechanical, petrochemical, and biomedical industries, to name a few. Several investigations have been carried out to study the coating performance developed through the PEO process in the past. This review attempts to summarize and explain some of the fundamental aspects of the PEO process, mechanism of coating formation, the processing conditions that impact the process, the main characteristics of the process, the microstructures evolved in the coating, the mechanical and tribological properties of the coating, and the influence of environmental conditions on the coating process. Recently, the PEO process has also been employed to produce nanocomposite coatings by incorporating nanoparticles in the electrolyte. This review also narrates some of the recent developments in the field of nanocomposite coatings with examples and their applications. Additionally, some of the applications of the PEO coatings have been demonstrated. Moreover, the significance of the PEO process, its current trends, and its scope of future work are highlighted.

## 1. Introduction

**Figure 1:**
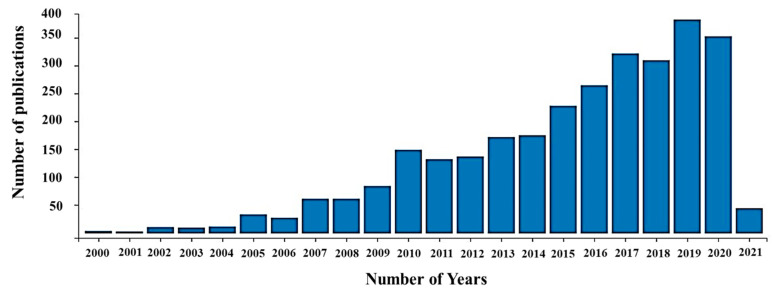
Number of papers published in the research area of plasma electrolytic oxidation treatment in the period from 2000 to 2021 (data taken from the web of science).

Plasma electrolytic oxidation (PEO) is a promising novel electrochemical surface treatment technique that can produce a thick, hard, and dense ceramic-like coating on aluminum, titanium, magnesium, and other lightweight alloy substrates. This process is also often termed micro-arc oxidation (MAO), plasma chemical oxidation (PCO), or anodic oxidation by spark discharge. PEO employs environmentally friendly weak alkaline as well as acidic electrolytes in which the oxide coatings are formed under the application of high electric voltages [1,2]. During this process, the thickness of oxide coatings grows in the range of tens to hundreds of microns on the substrate which can significantly improve corrosion resistance, wear resistance, and thermal barrier properties. The PEO coating process has been actively involved in research over a long period (Figure 1) and is considered a better approach for biomedical, electronic, aerospace, and automobile applications than other conventional surface treatment processes [3].

The plasma electrolytic discharge phenomenon was initially observed and recounted by Sluginov [4], and later, this process was further investigated by Güntherschulze and Betz in 1920 as a feature to develop electrolytic capacitors [5]. These phenomena were successfully studied in early 1970 by Brown and his co-workers and employed to produce ceramic coatings on Al alloy substrates in alkaline electrolytes [6]. They named this approach anodic spark deposition (ASD). Since then, researchers have further investigated this process, and after essential modifications that were made between 1980 and 1990, practical applications became feasible [7,8,9,10]. With the advent of commercial production and technology improvement, this process was then named the PEO coating process. Presently, this process is commercially developed and practiced by companies such as Keronite in the United Kingdom; Innovent, Cermanod, Meotec in Germany; and bioengineering based companies like Nobel Biocare in Switzerland; Keystone Dental in the USA; and Nano Prime in Poland to name a few.

PEO coating formation is a complex process as it involves three simultaneous operations: electrochemical reactions, plasma chemical reactions, and thermal oxygen diffusion reactions. The coatings obtained from these reactions usually comprise three-layer structures with a porous outer layer, a dense intermediate layer, and a thin inner dense layer.

**Figure 2:**
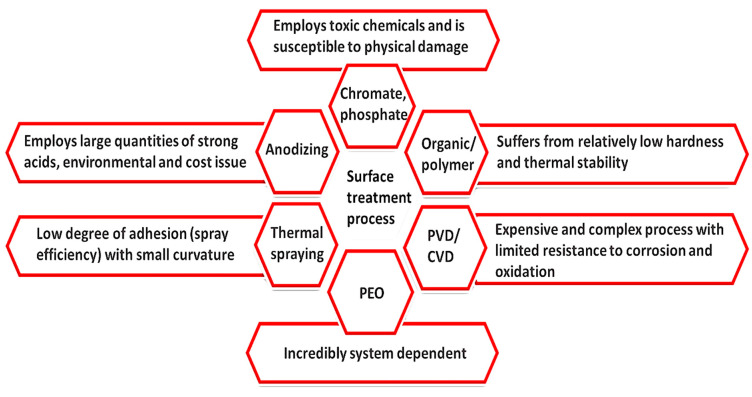
Comparison of common surface treatment techniques, a limitation for valve metals and alloys. Adapted from [12].

Numerous coating approaches have been developed for valve metals and their alloys. Examples of these coatings include chemical conversion coating, anodizing, electroless/electroplating, organic coating, laser surface treatment, vapor (physical vapor and chemical vapor) based deposition, and thermal/cold spray [11]. Most of these techniques use toxic or harmful chemicals to produce a coating on the substrate and require appropriate care and control during operation. The coating processes mentioned above might have some limitations with the substrate or base material, mainly caused by low adhesion forces, delamination of the coating surface, pores, and growth of cracks resulting in potential equipment failure. In contrast, the PEO is less complicated as it typically employs a non-hazardous and environmentally friendly solution to produce coatings, and achieves maximum efficiency oxide coating with lower costs and higher durabilities. A comparison of some of the common surface treatment techniques as well as their limitations is shown in Figure 2.

Anodization or anodic oxidation is a classical surface treatment technique implied for valve metals and their alloys to form a protective oxide layer [13]. In this process, a uniform voltage or current is employed between the two electrodes in an electrolytic solution to produce a thick oxide layer on the metal anode substrate. Anodizing was initially employed on an industrial scale during 1923 to safeguard aluminum-built seaplanes from corrosion [14]. The oxalic acid anodizing process was patented in 1923 (in Japan) and was employed mainly for architectural applications. The anodization process is generally carried out at a low operating temperature and high current density. Here the surface structure and chemical composition of the surface oxide film can be modified by tuning the parameters such as electrolyte concentration and composition, electrical parameters, and substrate composition.

PEO process is an advanced version of the anodization technique that employs higher potential to promote chemical, thermal, and plasma reaction on the substrate in order to form a thick, dense ceramic oxide coating. The PEO process also allows the formation of new surfaces with different colors and textures that eventually provide enhanced wear and corrosion resistance, thermal stability, dielectric property, better bioactivity, and biocompatibility [1,15]. A more comparable process to PEO is hard anodizing as it provides a thicker and more durable coating than normal anodizing. Table 1 summarizes some of the major differences between the PEO and hard anodization (HA) processes.

### 1.1. PEO Coating Mechanism

In a PEO process, varying the current mode between alternating current (AC), direct current (DC), unipolar, and bipolar current modes may lead to significantly different surface modifications. Primary parameters like electric current density, electric voltage intensity, pulse oscillation, and positive/negative duty cycle impact the PEO procedure and obtained coating attributes [17]. Moreover, these parameters also influence PEO process characteristics such as developed voltage breakdown, local melting and oxidation of the substrate, quenching, and recrystallization processes and in turn, significantly influence the coating’s microstructure and properties. In a PEO process, a wide range of voltages (95 V to 750 V) is employed along with an AC or DC electrical supply between the cathode and anode. On completing an electrical circuit, cathode and anode follow Faraday’s and Ohm’s Laws. Here, the metal substrate acts as a working electrode (anode) along with a counter electrode (cathode, generally formed of stainless steel or graphite) [18]. Both the electrodes are immersed in a weak alkaline electrolyte. The electrodes are then connected to an external electric supply in order to start the coating process. The electrochemical oxygen evolution and metal oxidation reactions occur at the anode during the PEO process (Equations (1) and (2)). In the process, anodic generation of oxygen takes place due to the high electric field, and oxygen anions (O^2–^) migrate towards the anode such that they form a metal oxide with the substrate. Depending on the substrate, electrolyte composition, and chemical activity, this could then result either in surface dissolution or formation of an anodic oxide film (Equation (3)). At the same time, intensive hydrogen gas evolution (Equation (4)) and cation reductions occur at the cathode surface (Equation (5)). Owing to the liberation of gas and discharge reaction, the obtained coating can be more porous compared to the oxide film from a hard anodization process.

The general equations involved in a PEO process are [19,20,21]:

2 H_2_O − 4 e^–^ → O_2_↑ + 4 H^+^ (1)

Me − *n*e^–^ → Me*^n^*^+^ (2)

*x* Me*^n^*^+^ + 3/2 *x n*·H_2_O → Me*_x_*O*_n_*↓ + *x n* H_3_O^+^ (3)

2 H^+^ + 2 e^–^ → H_2_↑ (4)

Cat*^n^*^+^ + *n* e^–^ → Cat^0^↓ (5)

where M depicts metallic material on which PEO is to be performed, n is the number of transferred electrons, and $e^{-}$ is the electron, and Cat is the cation.

**Figure 3:**
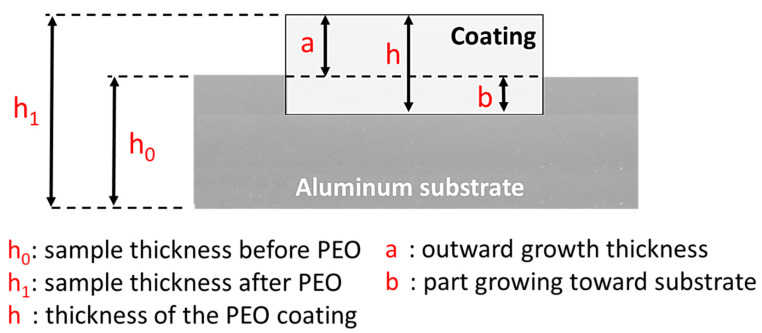
A schematic diagram explaining PEO coating formation on a 1060 Al substrate. Adapted from [40]. Copyright Elsevier, 2020.

Snizhko et al. [22] reported that the Faradaic process is associated with conventional hard anodization, but there have been circumstances where non-Faradaic phenomena were observed simultaneously along with the discharge reactions. It has been later highlighted that non-Faradaic processes are also involved in a PEO process, such as thermal disintegration of water. Apart from these two processes, the present research on PEO mainly focuses on two aspects. In the first aspect, research is being undertaken to better understand the fundamentals of the PEO process, such as electric discharge reactions, the liberation of gas [22], and acoustic emission [23,24]. The second aspect is concerned with different coating properties such as corrosion and oxidation resistance [25,26,27,28], coating wear property attributes [29,30], photocatalytic effectiveness [31,32,33], bioactivity [34,35], and thermal shock resistance [36,37]. All these aspects are important for the coating formed. The general mechanisms that take place during a PEO process are (a) formation of an oxide layer on the boundary between the metal and electrolyte, (b) increase in potential difference across two sides of the dielectric oxide layer, and (c) break down of dielectric oxide layer [38]. At the beginning of the oxidation process, an initial thickening of the oxide layer occurs outwards, followed by inward thickening of the oxide layer [39]. This process is illustrated in Figure 3.

The obtained oxide layer often results in a stiffer and crystalline structure as the electric discharges emit a considerable amount of heat, resulting in an increase of crystallization around the oxide layer [41]. Coatings subjected to PEO contain a high porosity level but are relatively more wear-resistant than anodized coatings [41]. The dielectric breakdown occurs between a thin oxide layer at the foundation of a deep pore in the coating. In a PEO process, higher voltage and AC result in intense sparks owing to micro-arc discharges that lead to the formation of break down oxide layers.

**Figure 4:**
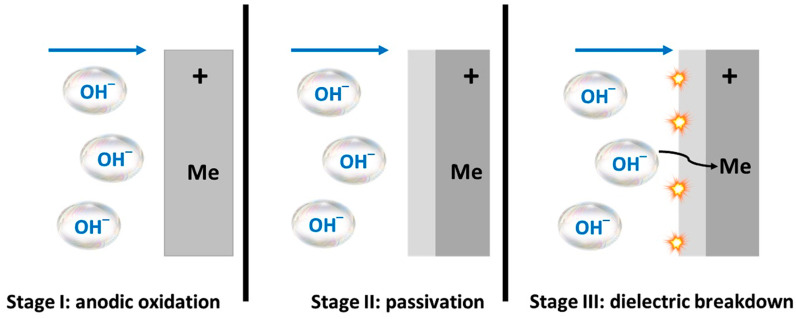
A schematic diagram explaining primary stages of an oxide layer generation in anodizing and PEO process. Adapted from [38]. Copyright IntechOpen, 2012.

There are some significant differences in the features of a PEO compared to an anodization process. The layer formed by anodization has a relatively uniform shape, and the thickness of the produced oxide layer is small due to the low electrical conductivity of the oxide layer. The developed layer passivates the metal surface during the anodization process and restricts its further growth (Figure 4: Stages I–II). On the other hand, in a PEO process, higher voltages produce intense sparking owing to micro discharge arcs that break down the oxide layer (Figure 4: Stages I–III). High temperatures and pressures develop in the discharge channels and create complicated phase transformation procedures which lead to the generation of a compact, thick, and hard layer that possesses resistance to abrasion and corrosion. Even though PEO layers have a comparatively higher porosity, they can efficiently protect the base metal against corrosion, since the pores formed by a discharge can be later healed by molten oxides due to high local temperature in the region of plasma discharge channels. Hence, PEO coatings are impervious to corrosive media.

PEO process is impacted by different parameters such as nature of substrate material, electrolyte constituents, the density of the current, type of current, voltage, frequency, duty cycle, additives, particles incorporated, coating time, and operational temperature [3,42,43,44,45,46,47,48,49,50,51]. Although this process has been widely used for materials such as aluminum and its alloys [50,52,53,54,55], and magnesium and its alloys [56,57,58,59,60], the focus of more recent research on PEO coatings are oriented towards other valve metals like titanium [61,62,63,64,65], tantalum [66,67,68,69,70], zirconium [71,72,73,74,75], and niobium and all their different alloys [76,77,78,79,80]. Moreover, there has also been considerable work reported on zinc modification by the PEO process [81,82,83,84]. Apart from the above-mentioned materials, a few studies have been carried out upon other less common metals such as hafnium [85], beryllium [86], and brass [87].

### 1.2. Processing Conditions Affecting PEO Process

**Figure 5:**
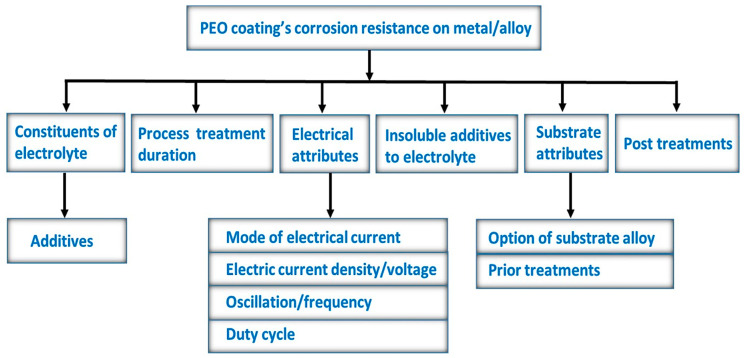
Processing conditions in corrosion resistance of PEO coatings. Adapted from [39].

During the coating process, various parameters influence the coating’s quality, such as electrolyte composition, substrate material, process time, voltage, and current type, i.e., AC or DC. In addition to the above-mentioned parameters, there are other factors that influence the coating such as gas bubbles formed, development of soft plasma discharges at the later stages of the process, electromagnetic radiation emitted which is also known as galvonoluminescence phenomenon, the temperature of the plasma, the rapid motion of dispersed particles in the electrolyte when an electric field is applied which is also known as electrophoresis, and energy consumed during the process [2]. The PEO coating morphology, microstructure, and phase constituents ultimately determine the coating’s performance. Some of the key parameters that play an essential role in a PEO coating’s corrosion resistance is shown in Figure 5. Therefore, in the next section, we will give a brief overview of the studies analyzing the influence of process parameters on PEO coatings.

#### 1.2.1. Impact of Electric Current

The type of power employed in a PEO process impacts the morphology of coating developed, the structure of the coating, rate of growth of coating thickness, the hardness of the coating, and magnitude of porosity [88]. The different modes of electric current, as mentioned earlier, that can be employed during the PEO process are DC and AC, with the unipolar and bipolar current for specific applications. Although DC can be used for coating purposes by the PEO process, the process becomes more effective when AC is used. DC is more useful in applications that need porous and thin coatings [89]. Additionally, the DC mode supports the production of coatings where the rate of formation of the oxide growth layer is relatively lower, leading to structures with higher porosity. This current mode gives less control and fewer tunable discharge characteristics, but if a pulsed DC mode is employed, then it will allow better control of discharge duration and possibly lower energy consumption [90,91]. DC application is also relatively cheaper and more convenient than AC. On the other hand, the benefit of employing AC mode is that it stops the polarization of the electrode and helps the process to be controlled by arc interruption [92]. It has been observed that bipolar pulse current modes produce higher and thicker coatings with superior corrosion resistance since it lowers the effect of high-intensity plasma discharges as well as high-temperature spikes when employed in a PEO process [88,93].

With respect to DC voltage modes, Stojadinovic et al. [94] explored its impact on the PEO process with zirconium (Zr) alloy in the presence of citric acid. They used optical emission spectroscopy (OEM) to characterize micro discharges during PEO. The study highlighted that the optimum choice of experimental parameters, like type and volume of electrolyte, plays an important role in determining the characteristics of the coating formed. Other factors such as temperature and electron density allowed for a more detailed understanding of the PEO process as well as the ongoing mechanisms. Zhao et al. [95] employed DC to refine the surface performance of few samples of Al-Zn-Mg-Mn-Zr alloys. This process was characterized by the formation of micro discharge sparks in four different stages. It was observed that in the first stage, there was the formation of a passivation film by a strong electric field, whereas, in the remaining three stages, the passivation film was produced by break down voltage. They found that the coating had a thicker layer at a lesser current density and with a lower number of inner defects. In contrast, high current density had a harmful influence on the composition and attributes of the coating. Sowa et al. [96] studied the influence of DC on the PEO coating of pure Zr in the presence of calcium acetate and calcium glycerol phosphate electrolytes. The process was carried out at 200, 300, and 400 V. It was observed that 200V resulted in the most corrosion-resistant coating without any porous structure. This type of coating was highly preferred for biomedical applications. When carried out at 300 V and 400 V, the process resulted in lower corrosion-resistant coatings, which is in contrast to the 200 V process. Further, the thickness and roughness of the obtained coatings were found to increase with increasing voltages. The impact of electrolyte constituents was negligible on the surface characteristics. Pitting resistance of zirconium was observed to have improved after the deposition of PEO coatings irrespective of the applied parameter conditions. In another study, Akbar et al. [97] analyzed the impact of the duty cycle on PEO coating thickness operated under unipolar DC power. It was observed that the coating thickness decreases with an increased duty cycle, and concluded that DC influences PEO coating in various ways, and further research can be carried out to modify the coating.

PEO coatings have been applied on different metal substrates with various electrolytes using AC mode [23,98,99,100]. Naeini et al. [101] investigated a biocompatible ceramic layer of TiO_2_ and hydroxyapatite (HA) nanoparticles deposited on pure titanium (Ti) using AC-based PEO coating. In the experiment, a titanium specimen was used as the anode and stainless steel as the cathode. Five different types of electrolytes with varying molar concentrations were employed in the experiments, as shown in Table 2. Initially, PEO coating was performed on Ti specimens in the existence of electrolytes without the addition of HA nanoparticles. Later, HA nanoparticles were added to the samples through the electrolyte. It was observed that coatings in the solution containing electrolytes 1, 3, and 4 resulted in better incorporation of HA nanoparticles due to pores forming during the PEO coating process. FE-SEM images from the experiment established that incorporation of HA nanoparticles was more noticeable in samples with electrolytes 3 and 4 compared to 1.

**Figure 6:**
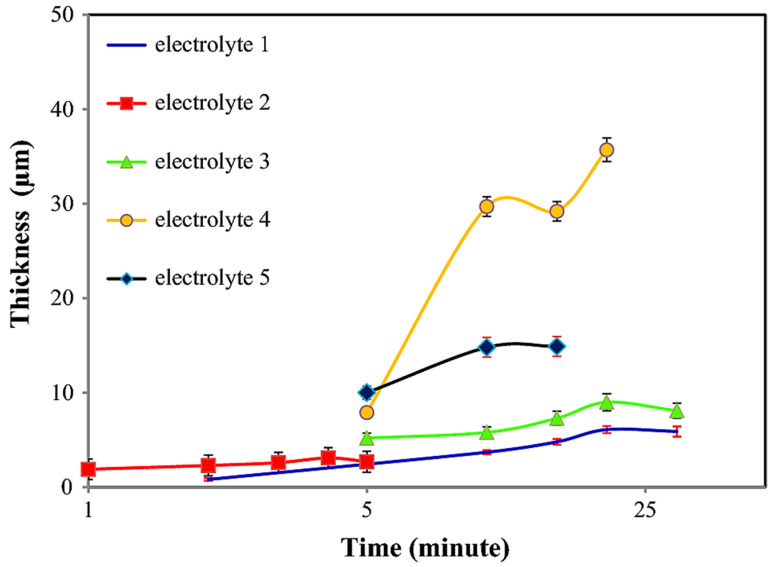
Comparison of coating thickness obtained from electrolytes versus time. Reproduced with permission from [101]. Copyright Springer Nature, 2019.

Among all the electrolytes, it was observed that electrolyte 4 generated the highest coating thickness. Furthermore, for all the electrolytes, after reaching a breakdown voltage, it resulted in a decrease in sample thickness. This could be credited to the fact of local heat generated during the PEO process and discharge formation impacted the sample’s thickness [102]. It was also observed that coated samples with HA nanoparticles experienced less corrosion than coated samples without HA nanoparticles. From the obtained results of this study, it could be concluded that the addition of HA nanoparticles significantly improves the material properties of the Titanium samples. Figure 6 highlights the variation of coating thickness produced for all the electrolytes with a gradual increase in time.

During a PEO process performed under AC, it has been observed that pores develop during the breakdown of the anode. These pores are repaired by fluidic oxides generated during a corresponding anodic pulse. Furthermore, the electrolyte near the specimen gets revived and fresh oxide layers formed are more uniform. Generally, 50–60 Hz sine-wave AC voltages between 100–600 V are employed for the PEO process [103].

It has been proposed that PEO performed by AC undergo the following steps [16,20,103]:

(a) A barrier oxide layer develops on the border between the metal and electrolyte throughout the first anodic half-period.
(b) The potential difference across both sides of the oxide layer increases with the advancement of the anodic half period.
(c) When the dielectric layer breaks down, it is followed by electric sparks. New volumes of electrolyte are incorporated in the metal surface during the break down until voltage is sufficient for new breakdown events, resulting in penetration and expanding oxide layers.
(d) Relaxation of metal and oxide layer and partial reduction of oxidized specimens occur throughout the cathodic half periods.
(e) Nucleation and annihilation of gas bubbles during the process affect the evolution of the oxide layer.

These mechanistic steps of PEO performed by AC mode are different from the anodizing process, which is already completed at stage (a) and does not go any further.

**Figure 7:**
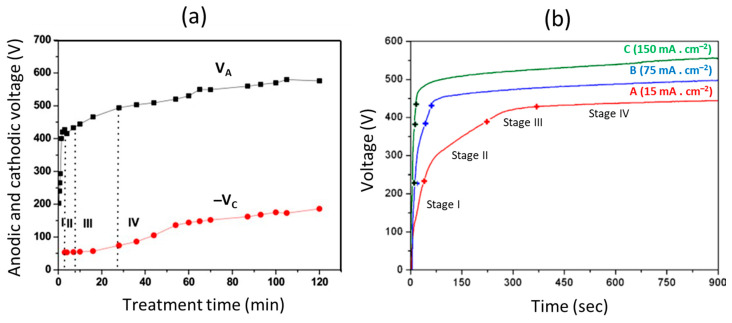
Graphs of voltage versus time for a PEO process pertaining to (**a**) AJ62 Mg alloy and (**b**) AM50 Mg alloy. Reproduced with permission from [12]. Copyright IntechOpen, 2014.

To understand the importance of the voltage characteristics and treatment time involved in a PEO process, two studies were compared on (a) an AJ62 Mg alloy employing bipolar current mode and (b) an AM50 Mg alloy operating DC mode, as shown in Figure 7 [12]. AJ alloys are magnesium-based die castable alloys possessing optimum creep resistance at higher temperatures. AM alloys contain aluminum and manganese, have good castability, and are employed in the automobile industry for dashboards, steering wheels, and seat frames. Four consecutive discharge stages were formed, which were unique for each of the studies. Stage I: This stage indicated the beginning phase of the PEO process, where quick electrochemical reactions on a nascent oxide film occur. Here, the breakdown voltage was not still obtained. Stage II: During this stage, the voltage rate was observed to drop and reduce, which was distinguished by the several discharge sparks traveling quickly over the entire specimen surface area. This phenomenon showed the beginning of the breakdown of the oxide layer along with the rise of temperature in the process, resulting in the melting of the substrate metal. Stage III: During this stage, there was a decrease in the increase of the voltage rate, which was distinguished by greater numbered but slower traveling discharge sparks. Stage IV: During this stage, the difference in the rate of voltage was observed to be slower than that of Stage III. Here, intense discharges form as comparatively more significant and prolonged sparks. For a few cases, these intense discharges could create irreversible destruction to the coatings in Stage IV. Figure 7 highlights (a) output anodic voltage (*V*_A_) and cathodic voltage (*V*_C_) as a function of the treatment time during a PEO process of an AJ62 Mg alloy employing a bipolar current mode, and (b) voltage versus treatment time of an AM50 Mg alloy employing DC mode.

**Figure 8:**
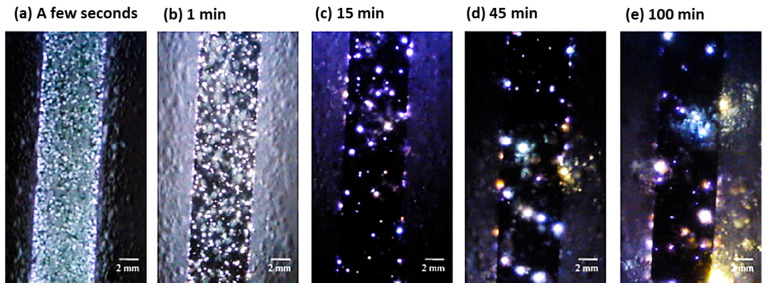
Snapshots during PEO coating depicting size and color of micro discharges produced at durations of (**a**) a few seconds, (**b**) 1 min, (**c**) 15 min, (**d**) 45 min, and (**e**) 100 min. Reproduced with permission from [104]. Copyright Elsevier, 2007.

In another study, Mecuson et al. [104] investigated a sequence of images from a high frame rate video of PEO coating employing AC mode applied on a 2214-T6 Al alloy as depicted in Figure 8. The images were analyzed for the rate of micro discharges with respect to size and color. During the initial stage of the process, a strong evolution of gas along with observable luminescence is observed at the alloy surface. This is accompanied by random sparks flashing over the entire surface of the alloy, as shown in Figure 8a. Figure 8b indicates the increase in the rate of micro discharges. Figure 8c depicts the gradual conversion of the micro sparks to micro arcs. Figure 8d shows the entire micro arcs regime. Figure 8e signifies the end stages of the PEO process resulting in irreversible destruction of the formed oxide layer.

#### 1.2.2. Impact of Electrolytes

The composition, nature (strong or weak), and concentration of electrolytes plays a vital role in determining the coating formed by the PEO process. Generally, the electrolytes employed in the PEO process are alkaline and weak. Electrolytes enable electric charges to move, which form a circuit and can adjust the electrical conductivity to be appropriate [105]. The aging of electrolytes is an essential factor of the PEO coating process [106]. Furthermore, the pH values of electrolytes play a significant role in the PEO process that can influence the microstructure and properties of the obtained coating.

Ghasemi et al. [107] generated PEO coatings on AM50 Mg alloys using KOH as an electrolyte with different additives such as silicate, phosphate, and aluminate, respectively. This alloy is widely used for automobile applications, but its application is disadvantaged with poor mechanical strength and corrosion resistance. It has been observed from the PEO process that the coating obtained from the silicate produced the highest coating thickness of about 8 μm, and the coating obtained from aluminate produced the least coating thickness of about 1 μm. Besides, the coatings formed from these electrolytes were found to possess various phase constituents. Additionally, PEO coating produced in electrolytes possessing the same additives, but different concentrations, can cause different features. It has been proven that increment in concentration of electrolyte generally produce thicker coatings and influences the coating’s porosity.

**Figure 9:**
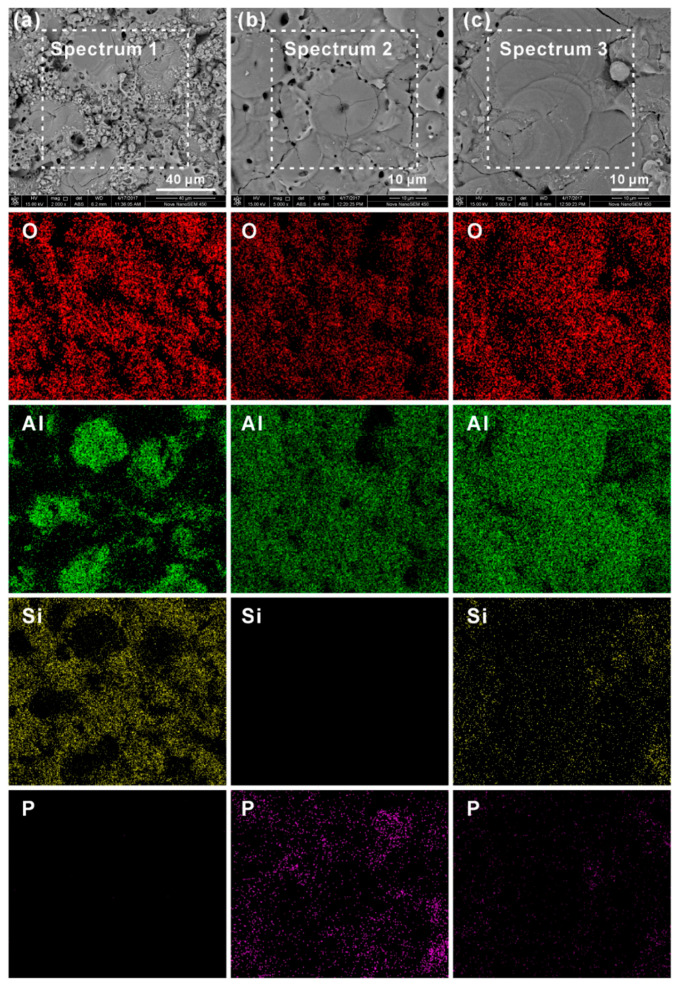
Depiction of surface morphology and distribution of elements for PEO coating produced by (**a**) silicate electrolyte, (**b**) phosphate electrolyte, and (**c**) mixed electrolyte of silicate and phosphate. Reproduced with permission from [40]. Copyright Elsevier, 2020.

Wang et al. [40] executed a PEO process on 1060 Al alloy in the presence of three electrolytes (perhaps different conductivity values), namely silicate, phosphate, and a mixture of silicate and phosphate. This alloy possesses superior electrical conductivity, corrosion resistance, and is widely used in electrical and chemical industries. It was observed that the breakdown voltage for the silicate system was 240 V, phosphate was 300 V, and with mixed electrolytes was 280 V. The fastest and highest coating thickness was observed for silicate, followed by phosphate, and finally, the mixed electrolyte system. The electrolytes were observed to influence the surface roughness of the coatings formed. The surface profile formed by silicate coating exhibited maximum fluctuation, followed by the phosphate electrolyte and then by the mixed electrolyte. Figure 9 highlights the surface morphology as well as the distribution of elements in the PEO coating prepared by the three electrolytes. All the PEO-coated surfaces were filled with pancake-shaped structures, and the individual pancakes possessed a tiny pore in the center, which was closely connected to the release of gas and discharge from plasma. It can be further observed from Figure 9a that the surface of the PEO coating produced from a silicate electrolyte, in addition to pancake structures, also possessed a large quantity of loose nodular protrusions, which were homogeneously distributed around the pancakes. Figure 9b highlights that in the phosphate electrolyte, pancake structures with closed centers were the major feature of the coating, although few pores were distributed surrounding the pancakes. Figure 9c highlights that in the mixed electrolyte, pancakes were the governing feature of the coating. However, there were relatively fewer pores as compared to the phosphate electrolyte formed coating. Some granular modules were observed surrounding the pancakes, but their shape and size were smaller than those produced by the silicate electrolyte. It could be concluded that type of electrolytes plays a vital role in the surface morphology and surface roughness of coating formed by PEO coatings.

**Figure 10:**
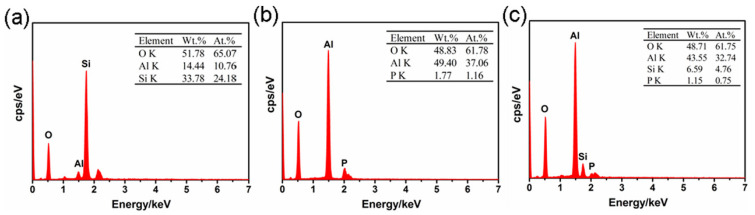
EDS spectra for (**a**) Si-coating, (**b**) P-coating, and (**c**) mixed Si-P coating. Reproduced with permission from [40]. Copyright Elsevier, 2020.

The EDS spectra from Figure 10 showed that for (a) Si- coating, the At.% of Si was relatively higher. However, for the (b) P-coating, the At.% of P was insignificant. In addition, for the (c) mixed electrolyte coating, both the At.% of Si and P were very small. The distribution of element O was comparatively uniform for all three types of coating.

**Figure 11:**
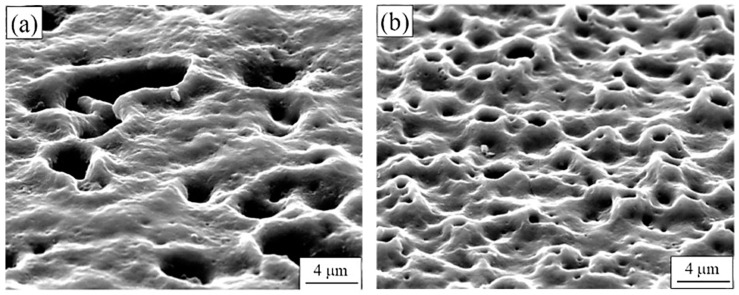
SEM images obtained after PEO process on a titanium sample in the presence of (**a**) K_3_PO_4_ electrolyte and (**b**) K_4_P_2_O_7_ electrolyte. Reproduced with permission from [108]. Copyright Elsevier, 2011.

Shin et al. [108] explored the effect of two different electrolytes on coating Ti by the PEO process. The sample was designed for biomedical investigation. The electrolytes employed were potassium pyrophosphate (K_4_P_2_O_7_) and potassium triphosphate (K_3_PO_4_), both of which are harmless to the human body. Both the electrolytes have the same pH. The PEO coating experiments were executed at room temperature to study the role of surface roughness and constitution of oxide films on Titanium. The coating was used to further study the tendency of biomimetic apatite formation in simulated body fluid (SBF). It was observed from SEM images (Figure 11) that K_4_P_2_O_7_ produced crater-like structures of micropores resulting in higher surface roughness. Surface with smaller pore size resulted in easier development of apatite layer. The XRD study from the same source showed that K_4_P_2_O_7_ produced more anatase phases that resulted in the quick growth rate of biomimetic apatite. Hence, it can be concluded that K_4_P_2_O_7_ was the better electrolyte for carrying out PEO coating on titanium pertaining to biomedical applications.

Ono et al. [109] analyzed the impact of electrolyte concentration on AZ31 Mg alloys exposed to PEO coating. The electrolytes used in this study were sodium phosphate, sodium silicate, and sodium aluminate. It was observed from the study that thickness and corrosion resistance of PEO films obtained for the same electric power supply increased with decreasing electrolyte volume despite the current density amongst all the electrolytes. Secondly, for all the electrolytes, it was noted that initial breakdown voltage increased linearly with the logarithm of decreasing electrolyte volume notwithstanding current density. Third, the chemical constitution of PEO films were similar and not dependent on electrolyte volume, current density, and reaction time. The corrosion resistance of PEO films was observed to increment as a basis of the logarithm of film thickness, regardless of electrolyte type and reaction conditions. Luo et al. [61] probed the impact of electrolyte concentration on Ti-6Al-4V alloy undergoing the PEO process. The electrolytes employed in this study were Ca(CH_3_COO)_2_·H_2_O, NaH_2_PO_4_·2H_2_O, EDTA, and NaOH. It was observed from the experiment that electrolyte concentration parameters have a negligible effect on the phase composition of the coating developed. The order of hierarchy of electrolyte concentration parameters on the efficiency of coatings was observed to be:

(a) Corrosion resistance of coating: EDTA > NaOH > Ca(CH_3_COO)_2_·H_2_O > NaH_2_PO_4_·2H_2_O;
(b) Strength of coating’s bond: Ca(CH_3_COO)_2_·H_2_O > NaH_2_PO_4_·2H_2_O > NaOH > EDTA.

Finally, in other experiments, the effect of electrolytes on PEO coatings, such as electron injection ability, improvement in adhesion strength, and thermodynamic stability was investigated in detail [110,111,112].

#### 1.2.3. Impact of Additives

The incorporation of additives plays a vital role in improving the properties of PEO coatings. The additives in the form of oxides, CNTs, or graphite have been found to improve the physical and mechanical properties of the PEO coatings. When additives are incorporated with the substrate, the resulting coating produces fewer defects and cracks. As a result, the corrosion resistance of the PEO coating is significantly enhanced. Table 3 has been formulated with names, substrate material, electrolyte employed, and properties of a few of them to explain the variety of additives employable in the PEO process.

**Figure 12:**
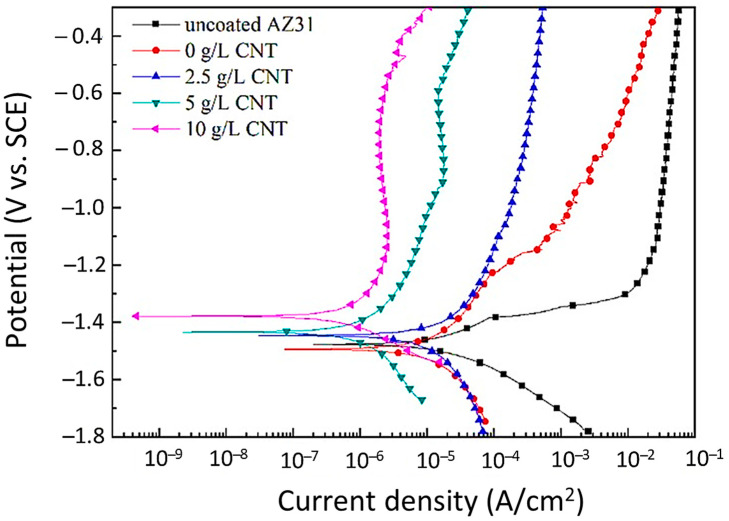
Potentiodynamic polarization curves of PEO coatings developed. Reproduced with permission from [129]. Copyright MDPI, 2018.

Zhao et al. [126] investigated the influence of various amounts of graphene oxide (GO) additives (1, 2, and 3 g/L GO) on AZ31 Mg alloy under Na_3_PO_4_·12H_2_O and KOH electrolytes, subjected to PEO coating. It was found that in all the PEO coatings, the addition of GO additives decreased the number of micropores. Electrochemical studies revealed that the addition of GO additives enhanced the corrosion resistance of the PEO coating. Two grams per liter of GO in the electrolytes produced the ultimate uniform coating, maximum carbon volume, and maximum corrosion resistance when compared with other electrolytes. Above 2 g/L GO in the electrolytes was noted to cause an increase in the number of micropores along with a decrease in the uniformity of coating microstructure. This resulted in lower corrosion resistance of the coating [126]. In another study, Li et al. [128] studied the effect of phosphate additives on magnesium–lithium (Mg-Li) alloy by the PEO coating process. The reason for choosing the Mg-Li alloy was that it has a wide variety of applications in various fields and are prone to corrosion. Two different kinds of electrolytes were considered in this study. The first electrolyte (coating A) used in this study was formulated from a solution of Na_2_SiO_3_, KOH, and KF in distilled water. The second electrolyte (coating B) was prepared from Na_2_SiO_3_, KOH, and KF in distilled water with the presence of phosphate additive, (NaPO_3_)_6_. The study highlights that the addition of phosphate additive helped in uniform morphology of the coating with higher thickness, hardness, wettability, and lesser defects of coating microstructure. The corrosion resistance of the coating was observed to improve with the incorporation of phosphate additive. It was noted that coating B could delay the onset of localized corrosion and impart long-term corrosion protection for 300 h. This good corrosion protection could provide a strong basis for the design of high-performance PEO coatings about Mg-Li alloys for usage in different applications. Junjie et al. [131] investigated the impact of a few additives on AZ31 Mg alloy examined for the PEO coating process under the presence of alkaline phosphate as a base electrolyte. Three different additives, Na_2_SiO_3_, NaAlO_2_, and K_2_ZrF_6_, were added in the same quantities (2.5 g/L) to the base electrolyte separately. Post PEO process revealed that the base electrolyte sample led to a smaller and abundant number of sparks on the coated sample. The sparks obtained from the addition of additives to the electrolyte were relatively larger since their final voltages were larger. The higher discharge sparks from additives were useful as more ions were entering the discharge channel and generating a higher amount of oxides. It was observed from the study that the base electrolyte produced a rougher surface on the coating. On the other hand, samples with additives were all found to make the surface of the coating smoother. It was noted that the K_2_ZrF_6_ additive produced a more uniform spark and showcased the best microstructure with fewer defects and a thick and consistent coating layer compared to all the samples. Moreover, the K_2_ZrF_6_ additives showed better corrosion resistance of the coating compared to other additives. Hwang et al. [129] investigated the effects of carbon nanotubes (CNT) additives (0, 2.5, 5, 10 g/L CNT) in 2 g/L KOH, 2 g/L KF, and 6 g/L Na_2_SiO_3_ electrolytes on AZ31 Mg alloy subjected to the PEO process [129]. The authors found that with increasing concentration of CNT, there was a gradual increase in the conductivity value of the samples. This could be justified by the fact that a higher concentration of CNT leads to higher conductivity. With an increase in CNT concentration, the coatings changed their color from lighter textures to darker textures due to the thickness of different phase compositions. To understand the corrosion behavior of the coatings, corrosion potential, corrosion density, and Tafel slope curves were employed, as shown in Figure 12.

**Figure 13:**
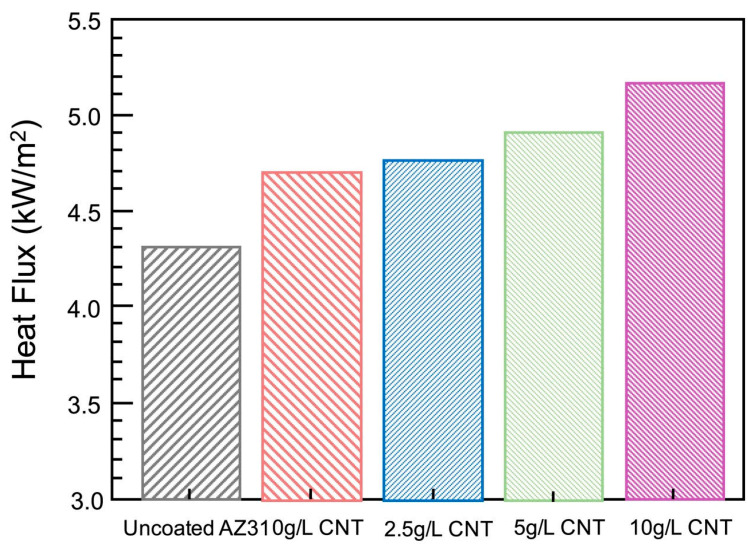
Heat flux of AZ31 Mg substrate and PEO coatings with various CNT concentration. Adapted from [129].

High potential and low current density signify enhanced corrosion resistance of oxide film. AZ31 Mg alloy has been observed to possess low corrosion resistance due to high corrosion current density and less corrosion potential. The outcome of the addition of the CNT additive was it boosted AZ31’s corrosion resistance. It was further observed that the sample with the highest concentration of CNT was noted to provide the best corrosion resistance. Figure 13 indicates that with the addition of CNT, there is a significant increase in the heat flux generated. The increase in the heat flux was due to the rise in thermal conductivity with a higher CNT concentration in the PEO coating. Higher heat flux resulted in increased emissivity/dissipation by CNT on the coating surface.

It could be deduced from this study that the addition of CNT additives on AZ31 alloy has multiple advantages, such as increased electrical conductivity of the electrolyte, reduced break down voltage, thin and dense PEO coating, superior corrosion resistance of PEO coating, more heat dissipation.

**Figure 14:**
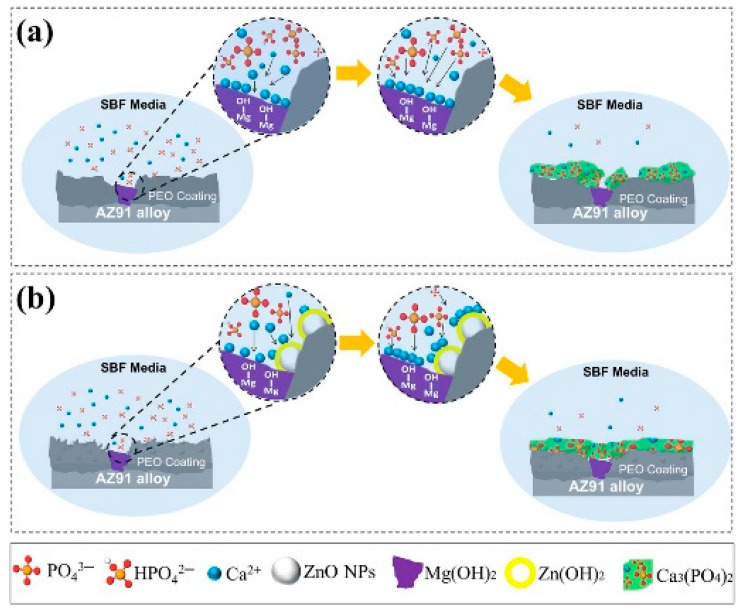
Illustration of the Ca_3_(PO_4_)_2_ layer formation on the PEO coating after being immersed in an SBF solution where (**a**) is without ZnO nanoparticles and (**b**) is with the incorporation of ZnO nanoparticles. Reproduced with permission from [130]. Copyright Elsevier, 2019.

Khiabani et al. [130] performed the PEO process for biomedical applications on AZ91 Mg alloy using ZnO nanoparticles to analyze the alloy’s corrosion resistance as well as in vitro biodegradation. The experiment resulted in the formation of a Ca_3_(PO_4_)_2_ layer of coating on the alloy. The coating was immersed in a simulated body fluid (SBF) solution for two weeks to analyze the bioactivity test. With an increase in ZnO nanoparticle intensity in the coating in the presence of SBF, it was observed that there was a higher growth of the calcium phosphate layer. Figure 14 depicts a drawing of the PEO coating formed in the absence and presence of ZnO nanoparticles.

**Figure 15:**
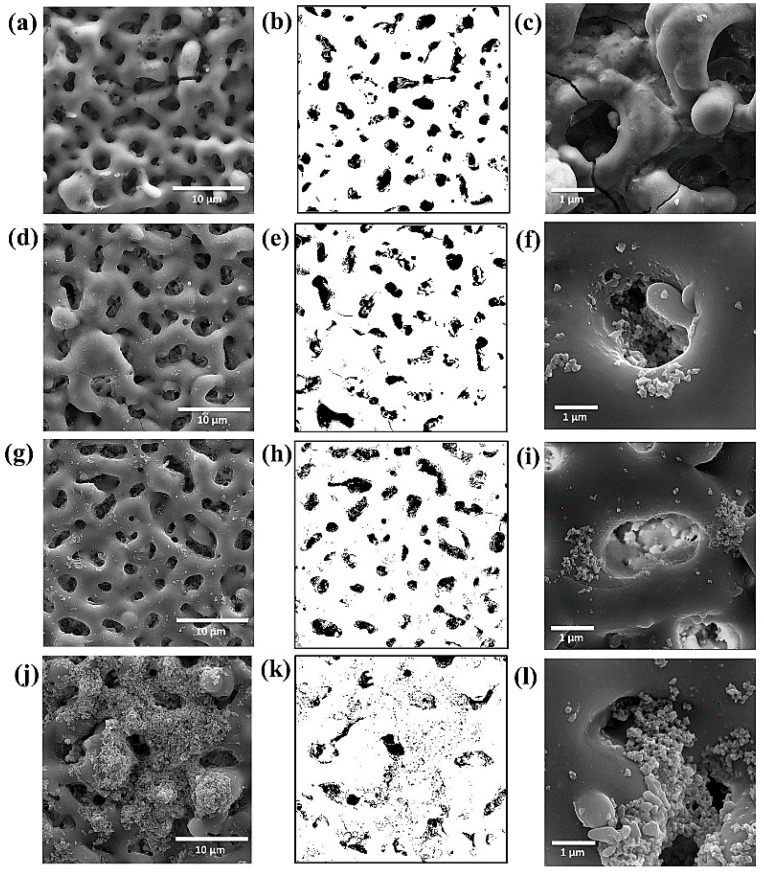
FE-SEM surface morphology for samples Z0 (**a**–**c**), Z1 (**d**–**f**), Z2 (**g**–**i**) and Z3 (**j**–**l**). Reproduced with permission from [130]. Copyright Elsevier, 2019.

In the same study [130], four sets of electrolytes, namely Z0, Z1, Z2, and Z3, respectively, were prepared. These electrolytes were comprised of tri-potassium phosphate tri-hydrate (K_3_PO_4_.3H_2_O) (5 g/L), potassium hydroxide (KOH) (2 g/L), and were treated with ZnO nanoparticles (0, 1.5, 3, and 4.5 g/L for Z0, Z1, Z2, and Z3, respectively). Increasing the concentration of ZnO resulted in the electrolyte sample containing higher pH as well as conductivity values from the experiment. The surface morphologies of the coating formed with the mentioned electrolytes are shown in Figure 15.

Incorporation of ZnO nanoparticles in the electrolyte decreased the coating thickness, surface roughness, and porosity of the coatings owing to the weakening of micro discharge arcs and obstruction of pores by ZnO nanoparticles. In addition, the existence of ZnO nanoparticles in the structure of coating enhanced the density, as a consequence of which coating’s resistance increased. It was further observed that the Z3 sample provided a complete layer of Ca_3_(PO_4_)_2_ on the entire coating and contained fewer pores. The generation of this layer produced the least sample mass loss as well as produced hydrogen gas. This attribute indicated enhanced bioactivity of the coating relative to other coatings.

#### 1.2.4. Nanocomposite Coatings

The incorporation of nanoparticles is an efficient technique to improve the quality of PEO coatings. These nanoparticles embedded in the structure of a coating allow PEO to produce composite coatings. Although a significant number of the embedded nanoparticles might enter the inner fragments of the coating, most of them remain diffusely distributed near the coating’s outer layer [132]. Hence the structure of the coating gets modified. The embedded nanoparticles in the coating structure not only enhance its corrosion and tribological characteristics but also improve its adhesion and hardness to the substrate. These nanocomposite coatings are employed in automobile, petrochemical, biomedical, marine, and electronic industries, to name a few. Arunnellaiappan et al. [133] incorporated *α*-Al_2_O_3_ and *m*-ZrO_2_ nanoparticles on AA7075 Al alloy in the presence of stearic and myristic acid by the PEO process, which resulted in the formation of nanocomposite coatings. These nanoparticles were found to increase the thickness as well as corrosion resistance of the coating. The addition of the stearic and myristic acid was observed to make the coating hydrophobic in nature and further improved the corrosion resistance. Sharifi et al. [134] employed PEO treatment on pure Ti by incorporating *α*-Al_2_O_3_ nanoparticles along with a non-toxic corrosion inhibitor, ketoconazole, to produce a dense nanocomposite coating. The presence of ketoconazole aided in the absorption of the nanoparticles made the coating dense, and reduced its porosity. The nanocomposite coating was observed to have improved hardness and wear resistance too. Atapour et al. [135] examined the influence of Ceria (CeO_2_) nanoparticles on AM50 Mg alloy to develop an aluminate-based PEO coating. It was observed that the incorporation of ceria reduced the porosity and roughness of the nanocomposite coating. In the future, research can be carried out to functionalize the nanoparticles to obtain superior coatings.

### 1.3. Primary Attributes of PEO Discharge

#### 1.3.1. Analysis of Radiation Spectrums Formed by PEO Plasma

Investigating the nature of plasma is a complicated endeavor owing to its temporary phase. Spectroscopic radiation studies have reported that the average temperature involved during the PEO process ranges from 3000 to 15,000 K [136,137,138,139]. Here, the electron densities generally vary from 10^21^ to 10^22^ m^–3^ [85,138,139,140]. PEO plasmas contain various groups such as the substrate, the coating itself, and the electrolyte employed [82,141,142,143]. The complete results about the various groups existing in the plasma depend upon the constitution of the substrate and electrolyte as well as on the electrical conditions that impact the formation and nature of plasma. From the literature, it was noted that a comparatively higher volume of the metal substrate is developed in the plasma, and the primary oxide of the coating is generated when the plasma cools down. After cooling, the metal oxide condenses into a liquid, quickly redistributing within the coating’s microstructure.

#### 1.3.2. Electric Discharge Characteristics

**Figure 16:**
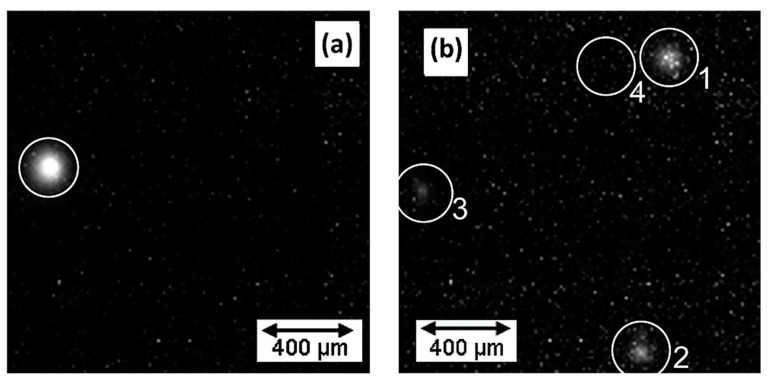
Discharge image captured by the high-speed camera when applied on (**a**) thick coating and (**b**) thin coating. Reproduced with permission from [144]. Copyright Elsevier, 2015.

Micro discharges occurring during the PEO process have lifetimes of the order of a few microseconds considering the incubation period to be around a few milliseconds to microseconds. Studies have been performed that highlight spectral discharges as combined data from many individual discharges. Nomine et al. [144] attempted to understand the individual localized discharge cascades formed during the PEO process on 6082 Al substrates by employing a high-speed video capturing device (acquisition rate of 180,000 frames per second). From the experiment, it was observed that the individual discharges occurred in cascades at a specific location, and each sequence comprised tens or hundreds of individual discharges. The individual discharges were distinguishable owing to their background light levels. The study highlighted that a sequence of discharges took place at a specific location for a thick coating, as shown in Figure 16a. On the other hand, for a thin coating, a series of discharges occurred at four different locations (Figure 16b).

**Figure 17:**
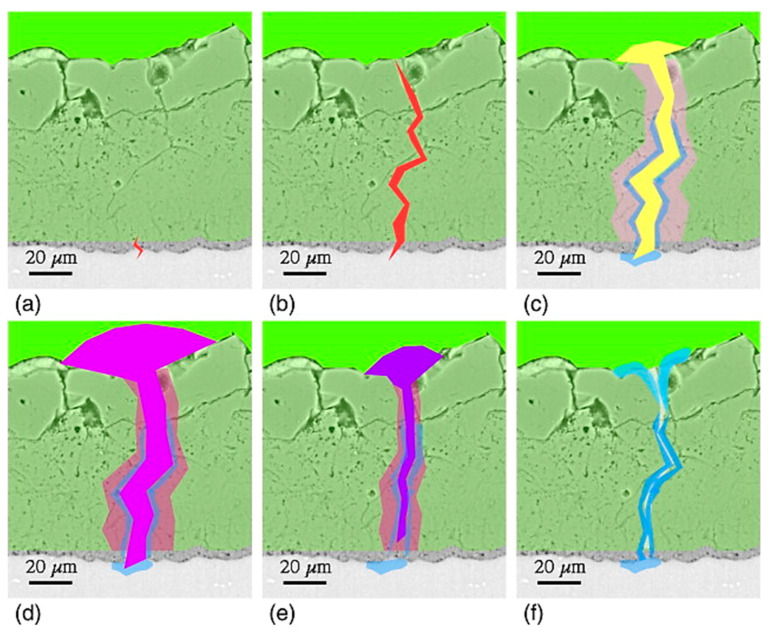
Description of events taking place during single discharge: (**a**) break down at beginning, (**b**) formation of plasma channel through coating, (**c**) beginning of bubble growth and generation of oxide, (**d**) enlargement of bubble and heating of adjacent region, (**e**) decrease of bubble area due to cooling, and (**f**) last stage signifying quenching and removal of liquefied oxide from discharge channel. Reproduced with permission from [144]. Copyright Elsevier, 2015.

It was further observed that a specific cascade happened in a well-defined location of the coating and consecutive light emission events took place having similar radii around the same point. As the thickness of coating increased, most of the characteristics remained unaltered, except light emission was noted to have a larger radius and higher intensity with thicker coatings. Figure 17 explains the mechanism of single discharge taking place in this experiment.

The duration of a cascade can often be too long (around a second), but in reality, during the PEO process performed by AC, the spark discharges develop only during the anodic part of the PEO process [145]. At this time, the voltage is above a threshold limit, which limits the time duration of a continuous chain to lower than a half-cycle period, which is a few milliseconds.

**Figure 18:**
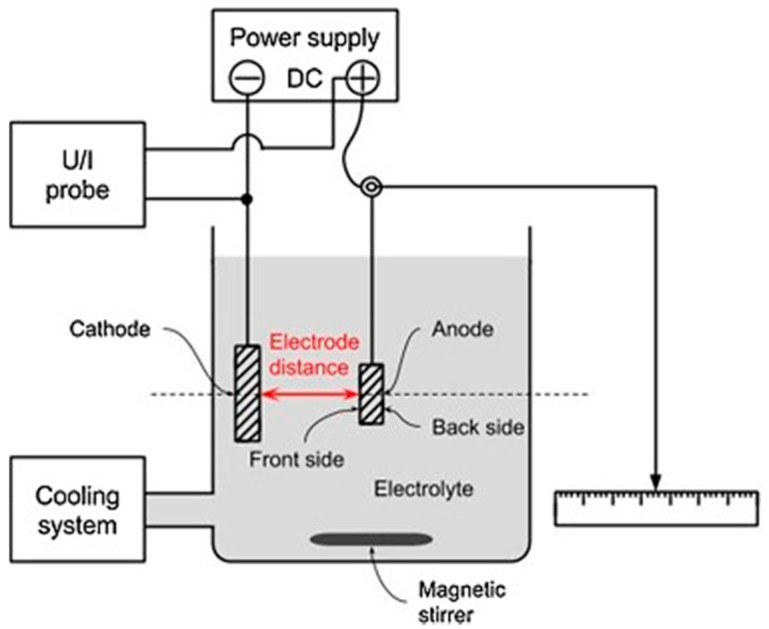
Sketch of the PEO experimental setup with stainless steel as a cathode and AM50 alloy as anode. Reproduced with permission from [151]. Copyright Elsevier, 2016.

**Figure 19:**
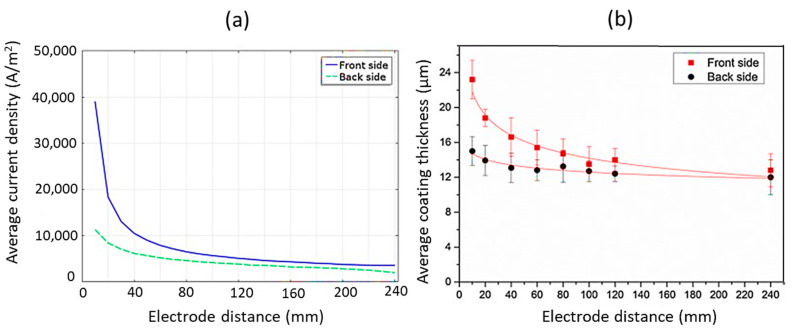
Graphs depicting (**a**) average current density on the front and back sides of the substrate versus electrode distance, and (**b**) average coating thickness produced against electrode distances of 10, 20, 40, 60, 80, 100, 120, and 240 mm for both front and back sides. Reproduced with permission from [151]. Copyright Elsevier, 2016.

Studies have been carried out to correlate external conditions of electric discharge characteristics and attributes of resultant coatings [146,147,148]. There have been other studies conducted to correlate the energy of single discharges with that of the entire PEO process [144,149,150]. Apart from these factors, physical factors such as the distance between electrodes have been found to play an important role in spark discharges. Ma et al. [151] carried out a PEO experiment on AM50 Mg alloys in an alkaline electrolyte having various electrode gap distances and uniform electric voltages. The setup of the experiment is shown in Figure 18 below:

During the experiment, the cathode and anode were kept apart at random distances of 10, 20, 40, 60, 80, 100, 120, and 240 mm. It was observed that with an increase in electrode distance, there was a nonlinear reduction in average current density across both the front and backside of the substrate, which implies a more and more uniform distribution of current, as shown in Figure 19a. The coating developed from 10 to 40 mm exhibited rough surfaces. Between 60 to 80 mm, the obtained surfaces were found to be smooth. Between 80 to 240 mm, the surfaces again were rough but with modified appearances. It was even observed that coating thickness decreased non-linearly for both the front and backside of the anode, as shown in Figure 19b. The front exhibited relatively higher coating thickness compared to the backside, irrespective of the electrode distance. The study further highlighted that coating’s elemental composition, surface morphology, and thickness were impacted by the electrode distance, but there was no influence on the phase composition. It could be concluded from the experiment that electrode distance does play a significant role in the PEO process.

### 1.4. Micro Structures Study of PEO Coatings

#### 1.4.1. Rediffusion of Oxide Layer Post-Collapse of Plasma

**Figure 20:**
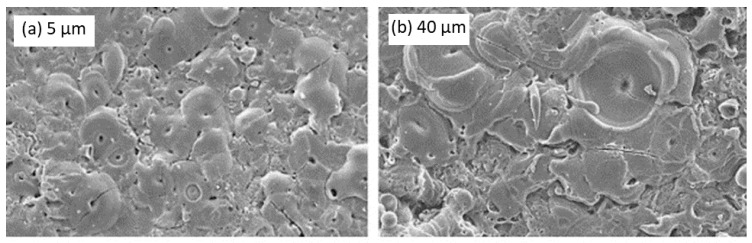
SEM micrographs of PEO coating on 6082 Al alloy developing coating thickness of (**a**) 5 μm and (**b**) 40 μm. Reproduced with permission from [152]. Copyright Elsevier, 2005.

New oxide layers are formed when each discharge occurs within the plasma after it cools down and crumbles. The liquid oxide gets inserted into the adjacent pores and cavities under high pressure. Due to high pressure, some of the liquid oxides enter the free surface, where they form volcano-like craters prior to solidification. These crater-like parameters are intrinsic to PEO coatings; however, their dimension and distribution can be significantly different [152]. These parameters are due to the liquid flow nature of the oxide during formation, as shown in Figure 20.

Prior studies have indicated that the flow was probably comprised of fluidic metal at the beginning. But recent studies have provided evidence that the flow was due to the oxide. The physical appearance can also confirm the difference between oxide and metal formed on coatings.

#### 1.4.2. Grain Structure and Phase Constitution of PEO Coatings

The microstructure of PEO coatings is comparatively complicated. Usually, quick solidification takes place at local zones. This quick process is characterized by several features, which include very fine grain structures and the existence of metastable phases. It has been revealed that Al alloy experiencing PEO coating contain γ, η, ε phases (in the coating) apart from the stable α phase [153,154,155]. The different phases obtained from the PEO coating of the Al alloys here were hexagonal close-packed (HCP) phase of α-Al_2_O_3_, monoclinic phases of γ-Al_2_O_3_, η-Al_2_O_3_, ε-Al_2_O_3_, and few amorphous phases. Similarly, the PEO coating study performed on Mg alloy suggest they contain HCP (α) and cubic (β) phases [156,157]. Ti sample coated by PEO generally has been found to possess anatase and rutile phases. Often these phases are accompanied by an amorphous material. Zr alloys treated with the PEO coating process have been observed to possess monoclinic, tetragonal, and Magneli phases [158,159]. Niobium (Nb) experimented with PEO coating contains pseudohexagonal and orthorhombic phases apart from the amorphous and crystalline phases [77,160]. Generally, heat treatment of PEO coatings to modify grain structures is a tough assignment because the span of temperature required to create significant changes for the oxides would usually be nearer to the melting temperature of the metallic substrate [41].

#### 1.4.3. Importance of Porosity and Graded Structures for PEO Coatings

The majority of PEO coatings are porous, possessing complicated structures and a broad range of scales [161,162]. Porosity levels and characteristics are associated with the way that discharges form. Research is being carried out to develop more uniform and superior pores from the PEO process [163,164,165]. It has been known that rough pores are usually not beneficial. However, in many circumstances, like the employment of PEO coatings in biomedical and photocatalytic applications, the existence of pores in coatings is advantageous, especially where a higher surface area is necessary [39,41]. The presence of pores in a PEO coating often leads to the mechanical stability of the coating, which also helps in reducing its stiffness. From a few studies, it has been reported that owing to the presence of pores in PEO employed coatings. There is a considerable decrease in Young’s modulus of the coated material present with pores when compared with the fully dense material [152,166]. This decrease in Young’s modulus increases relative to the strain tolerance of the coating.

Apart from the presence of pores in coatings, research is being done to develop coatings with composite or graded structures to enhance their performance. For example, sealing of the coating surface by some procedure might result in better resistance to the entry of corrosive electrolyte fluids without compromising the mechanical advantages of pores in the coating. It is hard to produce such sealed surfaces after PEO treatment; however, research is being carried out to develop such processes. These new structures include phase gradients of the coating [167,168], the introduction of layers of the polymer [169,170], employment of different kinds of composite coatings [171,172], and employment of metal-cored ceramic fiber networks [173].

## 2. Mechanical Attributes of PEO Coatings

### 2.1. Tribological Attributes

PEO coatings are essential for numerous surfaces that need resistance to sliding or abrasive wear. PEO coatings have been found to firmly adhere to substrates and possess lesser stiffness. As a result, strain energy release rates of this coating are lower. Although they possess lower stiffness, PEO coatings are relatively harder compared to the coatings obtained by anodization. Here, tribological studies play an important role in PEO coatings and are explained in the next section with few examples.

**Figure 21:**
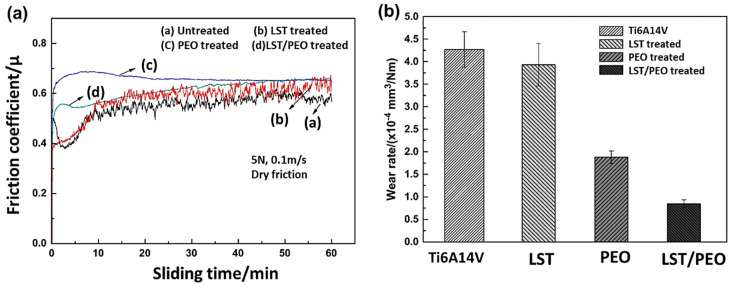
Variation of (**a**) friction coefficient and (**b**) wear rate. Reproduced with permission from [174]. Copyright Elsevier, 2015.

Qin et al. [174] carried out the PEO process on laser surface textured (LST) Ti_6_Al_4_V alloy deposited with MoS_2_ solid lubricant. Different samples that were considered for this process were untreated Ti_6_Al_4_V, LST treated Ti_6_Al_4_V, PEO treated Ti_6_Al_4_V, and combined LST, PEO treated Ti_6_Al_4_V. Under identical input conditions, LST, PEO treated samples exhibited the higher friction coefficient (COF), and untreated samples exhibited the lower COF (Figure 21a). When studying the wear rate for the same experiment under identical input parameters, the untreated sample exhibited the highest wear rate, and LST, PEO treated sample exhibited the lowest wear rate (Figure 21b).

**Figure 22:**
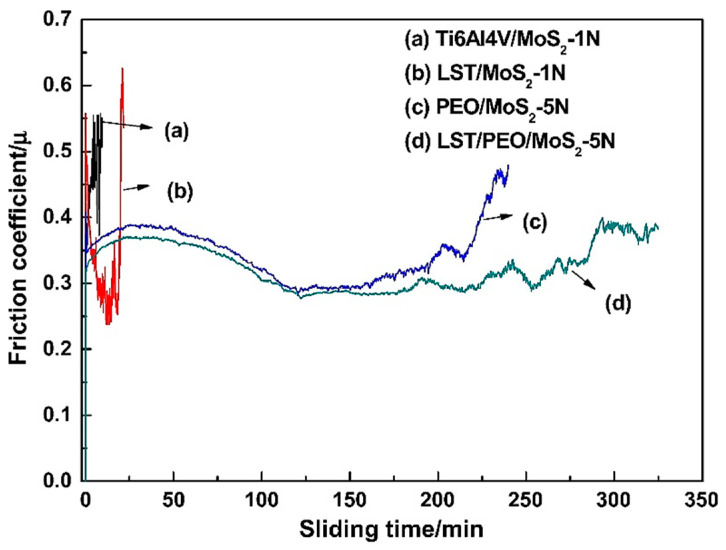
Friction versus sliding time graph for samples with addition of MoS_2_ additive. Reproduced with permission from [174]. Copyright Elsevier, 2015.

The addition of MoS_2_ solid lubricant on the samples resulted in a considerable reduction in the COF at the end states of the LST, PEO treated sample compared to other samples as shown in Figure 22.

**Figure 23:**
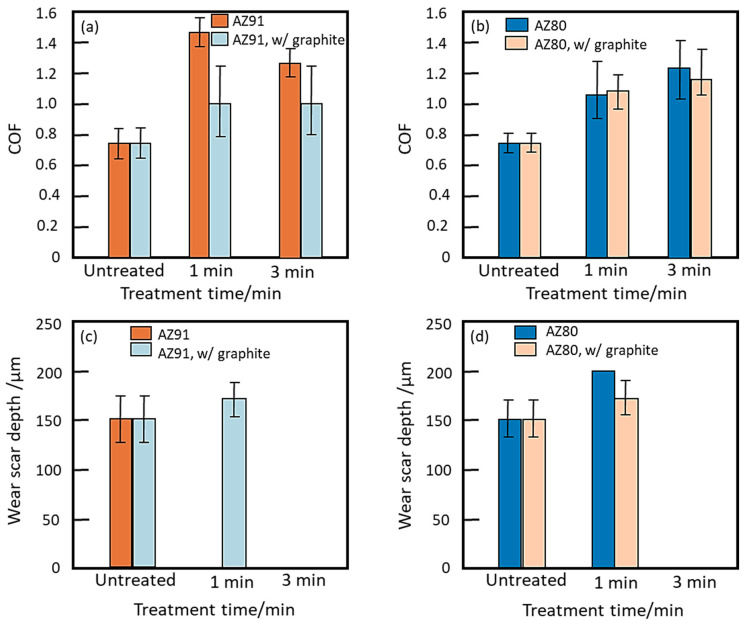
Bar charts comparing (**a**) COF for AZ91 with and without graphite, (**b**) COF for AZ80 with and without graphite, (**c**) wear scar depth of AZ91 with and without graphite, and (**d**) wear scar depth for AZ80 with and without graphite. Adapted from [175]. Copyright Elsevier, 2018.

Pezzato et al. [175] investigated the tribological action of PEO coatings with and without graphite nanoparticles on AZ91 Mg and AZ80 Mg alloys. Dry sliding tests were executed on both alloys with and without graphite additives for two different treatment times of 1 min and 3 min, respectively, against a steel counter surface. The COF for AZ91 with and without additive was highest for 1 min treatment time. This was attributed to the abrasive interaction of the hard and rough PEO treated AZ91 sample against the counter surface steel (Figure 23a). On the other hand, for AZ80 alloy with and without graphite, maximum COF was observed for a treatment time of 3 min (Figure 23b). When analyzing wear scar depth, both AZ91 and AZ80, with the presence of graphite, exhibited the lowest scarring (Figure 23c,d).

The extension of treatment time reduced the wear scar depth for both AZ91 and AZ80 alloy samples. Moreover, the addition of graphite considerably reduced COF for AZ91 alloy.

**Figure 24:**
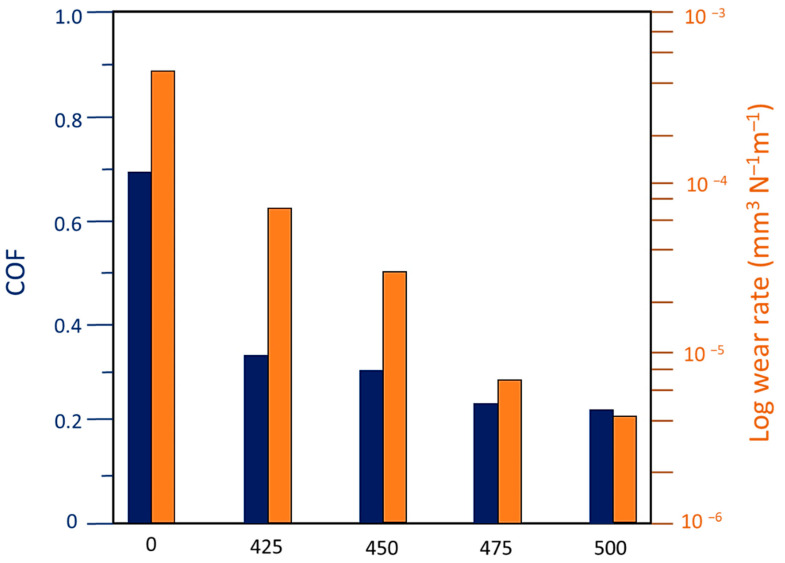
A graph comparing average friction coefficient and wear rate when sample experience different voltages. Adapted from [176].

Barati et al. [176] performed PEO coating on 7075 Al alloy in a DC mode. The tribological properties of the coating samples were analyzed by conducting a dry sliding test against tungsten carbide (TC) balls with a pin on a disk machine. The experiment was carried out with DC voltages of 425, 450, 475, and 500 V. Uncoated 7075 Al produced the highest friction coefficient. There was a gradual decrease in the friction coefficient as the voltage kept on increasing for the coated alloy (Figure 24).

It was assumed that the surface roughness (*R*_a_) value was highest for the sample treated with 500 V and least for the sample treated with 425 V. Hence it was expected that the sample treated with 500 V was supposed to exhibit the highest friction coefficient. In contrast, the experimental results showed that the 500 V treated sample had the least friction coefficient. The reason for this unexpected result was the evolution of hard phases along with the development of a higher tetragonal zirconia phase in the coating.

### 2.2. Impact of Fatigue Loading

Resistance to cyclic fatigue loading was influenced due to the evolution of cracks and the initial period of crack growth. Since these phenomena occur at the surface, it is crucial to analyze whether PEO coatings would increase or decrease resistance to these surface developments. Corrosion can also impact these surface development processes. There are often residual stresses in PEO coatings, which are compressive in nature and are relatively low. The loss of compressive stresses in the actual metal would result in degradation of the surface, which is not beneficial. Hence preprocessing of surfaces before carrying out the PEO process is important.

Winter et al. [177] performed the PEO coating process on 6082 Al alloy to understand the mean stress sensitivity of the alloy’s fatigue life. 6082 Al is a medium strength alloy with wonderful corrosion resistance and is used for transportation applications. Previous studies have shown that PEO-treated Al alloys exhibited poor fatigue performance compared to uncoated alloys. With the enhancement of coating thickness, there is a further decrease in fatigue performance of PEO coated Al alloy. The reduction in fatigue strength could be due to the porous nature of the oxide film that initiated early crack propagation. Three different applied load ratios (*R*) of −1, 0.0, and 0.1, respectively, were employed to investigate the fatigue performance of the Al alloy. The applied load ratio is defined as the ratio of the minimum and maximum loads considered during the process of fatigue loading. During the experiment, the PEO process time was reduced to obtain a thin film coating that would reduce the negative effect on fatigue strength. The maximum stress that can be applied on a material for a certain number of cycles without fracture is called its fatigue strength. After completion of the process, there was a significant decrease in fatigue strength pertaining to the PEO treated samples for all three load ratios. The PEO process was noted to nullify the mean stress impact on the fatigue life of the coating. Other studies have been noted where a decrease in fatigue life was reported for other Al and Mg alloys [178,179,180]. However, for the PEO process carried out on Ti_6_Al_4_V alloy, no change in fatigue life occurred [181]. After completion of the PEO process, residual electrolytes remained in the coating. If this residual electrolyte is not flushed out completely, corrosion may begin. Hence utmost care must be taken while carrying out the process and verify each step. On the other hand, Keronite company’s PEO treatment provides a dense and uniform oxide layer with fine-grained microstructure, thereby improving the fatigue properties of the material.

### 2.3. Residual Stresses

The coating generated from the PEO process gets repeatedly restructured by the micro discharges, hence, the residual stresses formed during this oxidation process get continuously lowered in magnitude. In addition, the comparatively low stiffness of PEO coatings causes hindrance to the creation of high stresses. But in a few cases, noted from literature studies, high residual stresses have been reported [90,168,182]. However, there are dissimilarities in these results. These high residual stress values were obtained from X-ray diffraction graphs. Application of this technique on PEO coatings is a difficult job because the microstructures of the coating contain very small-sized grains, possess various phases, and there are chances for considerable modification in coating microstructure. To overcome this limitation, a new technique called curvature measurement was employed. In this technique, it was found that general stress levels in PEO coatings were approximately 50 MPa for Al and 150 MPa in Mg [183].

## 3. Impact of Environmental Conditions

Various environmental factors influence the coatings developed by the PEO process. A few of them are explained below.

### 3.1. Impact of Corrosion

PEO coatings are mainly used to improve the corrosion resistance property of specific materials [184,185,186]. Improvement in corrosion resistance property is more significant for Mg alloys since they are more susceptible to corrosive degradation in the absence of surface treatment [117,187,188]. The main benefit of employing PEO coatings is that they rigidly adhere to the surface while other coatings generally do not. Corrosion resistance of coatings can be refined by customizing electrolyte composition so that external particles can be incorporated in the coating to enhance its performance [41]. It will also be necessary to ensure that any fluids do not easily enter the coating, hence, different types of surface sealing processes need to be employed.

### 3.2. Thermal and Optical Properties

**Figure 25:**
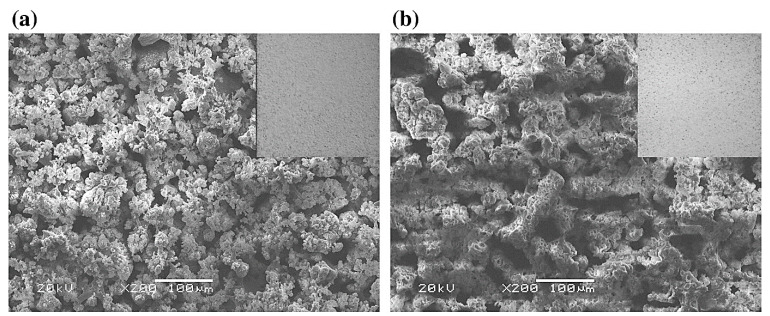
SEM micrograph of coating (**a**) before thermal shock test (**b**) after thermal shock test. Reproduced with permission from [192]. Copyright Elsevier, 2014.

Considerable interest has been generated amongst researchers to customize the thermal and optical properties of PEO coatings. Generally, metals undergoing the PEO process are good thermal conductors, but PEO coated structures in the metal and alloys exhibit low thermal conductivity. Often, these coatings act as thermal barriers [189,190,191] due to their increase in thickness. Thermal control coating is a traditional technique to balance the temperature of the surface by adjusting the absorption emissivity ratio of the coating surface. There are mainly two kinds of thermal control coatings (a) low absorptance emissivity ratio coating and (b) high absorptance emissivity ratio coatings. PEO coatings are observed to possess a high absorptance emissivity ratio. PEO process is a promising prospect for the development of thermal control coatings. Yao et al. [192] carried out the PEO process on Ti_6_Al_4_V alloy in the presence of an alkaline electrolyte. The coatings exhibited high emissivity and low absorbance properties under optimum working parameters. Since the developed coatings are expected to work under extreme conditions, they were put through thermal shock tests. The coating surface before and after the thermal shock test was almost the same, and there was no significant change in the thickness of the coating. However, the pore size of the coating slightly increased due to spalling (Figure 25) and there was an increase in surface coating roughness. The obtained coating was thermally stable and possessed good adhesion to the substrate. The crystallization of the coating slightly improved, and there was not much variation in its emissivity and absorbance properties.

Further interest aims at customizing the optical properties of PEO coating according to the individual application requirements. Optical properties pertaining to absorption in the visible spectrum are of greater interest since they decide the color of the coated surface. PEO experiments have generated yellow [65], green [193,194], grey [195], red [196], blue [197], black [198,199], and other coating colors. A study has been conducted to fabricate a porous and rough ceramic coating that could reflect active or passive infrared rays [200]. It is expected in the future interdisciplinary research will be conducted to produce superior coatings from PEO.

## 4. Applications of PEO

### 4.1. General Applications of PEO

PEO coatings have been widely used in various consumer industries such as biomedical, aerospace, electronics, automobile, and painting. Nowadays, PEO coating is employed to treat the surface of cell phone cases [201]. Table 4 shows some of the alloys and their field of application. PEO process has often been employed to develop an optimum keying surface for more surface treatment which can be observed in applications like a painting. This application is typically employed on Al alloys [53]. By customizing porosity, PEO treatment can increase the sticking properties of color paints, sol-gel coatings, powdered coatings, and form duplex coatings possessing superior properties. Generally, the thermal conductivity of PEO coatings is low, but under thermal cycling tests, PEO coatings exhibit better performance in the range of −40 to +100 °C. Therefore, PEO coatings can be employed in conjunction with thermal cycling tests to enhance the thermal shock resistance of the coating [189]. The PEO process carried on 6061 Al alloy reduced wear rate almost by a factor of 30 when compared to anodizing on 6061 Al alloy, which could minimize wear rate only by a factor of 2 [202]. This provides substitution of a few components that can now be subjected to the PEO process, especially on Al alloys which will lead to the lessening of fuel consumption from automobile and aerospace industries. Enhanced coating performance obtained from the PEO process has been employed to manufacture machine parts related to the aerospace, automobile, gas, and oil industries [203]. The inside components of a spacecraft are coated with flat absorber coatings for optimum radiation purposes. These coatings absorb most of the incident energy to optimize the temperature inside a spacecraft. PEO process is widely used to prepare functional ceramic coatings on aluminum, titanium, and magnesium alloys used in spacecraft. However, a limited study is available on the PEO process to produce thermal control flat absorber coatings required for spacecraft components. Pillai et al. [204] performed a PEO process on vanadium sulfate additive on 6061 AA aluminum alloy in a silicate-based electrolyte to produce a flat absorber black PEO coating. The study found that a uniform dark black coating was formed, possessing the high emittance and absorptance value required for an optimum flat absorber. The vanadium sulfate was noted to produce the black color of the coating as well as improve the growth rate of the coating significantly. Additionally, the PEO process is being employed to obtain electrically insulated coatings possessing good dielectric strength meant for electrical equipment and electronic components. PEO process is widely used in chemical industries owing to the resistive nature of PEO coatings when exposed to aqueous surroundings. These coatings are also resistant to strong acids and strong alkalis [12]. PEO coated Ti and Mg alloys are widely used in biomedical industries for their biocompatible PEO coatings.

### 4.2. New Combination Technologies Associated with PEO

PEO process is often inefficient for ferrous materials and produces a porous, thin coating for magnesium alloys. Hence to overcome these disadvantages, Martin et al. [222] investigated the duplex treatment of merging cold-spray (CS) deposition process with PEO. Initially, they performed a 1050 Al powder cold sprayed coating on an S235 steel and EV31 magnesium sample. Later these samples were experimented with the PEO process without incorporating any additives. It was observed from the study that the growth kinetic of the PEO oxide layer formed on the coating improved by a factor of three relatives to a single-step PEO process. Kozelskaya et al. [223] explored the combination of the PEO process with the radio frequency magnetron sputtering (RFMS) process. In this study, the PEO process was initially performed over Ti_6_Al_4_V alloys for biomedical purposes. Then, an upper layer of calcium phosphate (CaP) was deposited over the PEO coating by the RFMS process. The study found that the RFMS process allowed better integration of CaP to enhance bone growth and at the same time modified the surface texture into a spongy surface which would allow good bone growth structure. The dual coating resulted in multileveled roughness, enhanced Young’s Modulus and Ca/P ratio beneficial for osseointegration. Wierzbicka et al. [224] investigated a flash-PEO process performed on AZ31B Mg alloy in the presence of different electrolytes to develop an environmentally friendly coating that can be a substitute for traditional chromate coating. This alloy, due to its low weight, is used in the aircraft manufacturing industry. Flash-PEO is a relatively quick process that can help produce less costly coatings with optimum thickness and corrosion resistance compared to toxic chromate conversion coatings. The obtained coating exhibited enhanced corrosion resistance, self-healing properties, and good wet and dry adhesion to paint surfaces. The study from various researchers has found that the PEO process reduces the fatigue working of aluminum alloys. To overcome this limitation, Ye et al. [225] explored the synergism of a cold working treatment process and a hard coating to improve the fatigue resistance, wear, and corrosion resistance of a 7A85 Al alloy. This alloy is one of the most key materials required by the aviation industry. They employed shot peening (SP) process combined with the PEO process to study the corrosion fatigue nature of this alloy. The SP process that was performed as a pretreatment for the PEO resulted in the generation of residual stresses that could limit the intergranular corrosion. Additionally, the combination of the SP and PEO process was observed to increase the undesirable notch effect or crevice corrosion on the coating. Hence to reduce the notch effect, the surface underwent a polishing process immediately after executing the SP process. This not only reduced the notch effect but also greatly improved the fatigue and corrosion resistance of the coating. Titanium alloys used in alkaline mediums such as seawater environments are more susceptible to corrosion. Although research has been performed employing the PEO process on titanium alloys, less study is related to restoration of PEO coated titanium alloys. To overcome this limitation, Mashtalyar et al. [226] created a composite coating formed on already oxidized Ti alloy by combining the PEO process with the fluoropolymer treatment process. The PEO-coated titanium alloy was immersed in a fluidic PTFE dispersion. The incorporation of PTFE resulted in the sealing of pores for the composite coating. The coating showed a three-fold increase in wear resistance and had hydrophobic characteristics. This created a composite coating on the titanium alloy. Aluminum alloys used in aerospace applications are susceptible to fretting wear, especially in critical working environments. To address this issue, Lin et al. [227] developed a duplex coating consisting of multiple combined solid lubricants, also known as a chameleon, on a 6082 Al alloy undergoing fretting tests. The developed coating efficiently reduced fretting COF and enhanced the wear resistance of the base alloy. Additive manufacturing (AM) is a fascinating technique for developing individually designed objects. This process has been employed on alloys such as AlSi10Mg, F357, and AlSi12. The scope for printing AM alloys such as AlSi12 has some disadvantages like the formation of a less-coarse structure and growth of solidification cracks. To overcome these limitations, Rogov et al. [228] employed the PEO process on 3D printed AlSi12 alloys. It was observed that the coating obtained from this process possessed a hardness level five times higher than the base alloy. Additionally, the coating provided a uniform film on the base alloy surface for enhanced protection. Non-biodegradable plastics are abundant in supply and find different industrial applications [229]. However, when attempted to join with metals by traditional joining methods, to form new light-weight metals, they are often susceptible to degradation. Hence PEO process has been employed as an alternative to make adhere non-biodegradable plastics with metals effectively.

## 5. Scope of Future Work

The PEO process is a multiple-event process. There are several parameters involved in the process, most of them related to the substrate composition, electrolyte composition, electrical power supply, operational pressure, and temperature. These parameters play a vital role and influence the physical properties of the coating, such as microstructure, pore size, pore roughness, color, texture, hardness, and surface area, respectively. Hence there is an opportunity to modify these parameters to create superior coatings. One major area where further studies are necessary is to examine the parameters, microstructure, and properties interdependence. Additional studies from plasma formation, electrochemistry, electrical engineering, fluid mechanics, and heat transfer could help get a better understanding of parameters and coating microstructures and their related properties. PEO coatings are brittle in nature. Steps to overcome this limitation will enhance its material property. External and environmental conditions should also be investigated to be able to produce optimum and superior coatings.

## 6. Summary

The PEO process has been a standard technology to develop better surfaces for many years. There has been a significant increase in its application, and it possesses many special characteristics different from other surface treatment procedures. To improve the coating obtained from the PEO process, this review helps to understand the mechanisms involved in this process, the processing conditions that impact the process, the main characteristics of the process, the microstructures evolved from the coating, the mechanical attributes of the coating, and the impact of environmental conditions on the coating process.

Here, the influence of electric current on the coating is explained with state-of-the-art examples. The importance of the electrolytes employed in this process is discussed in detail. Additionally, the variation of additives that can be employed with the PEO processes are enlisted along with their functional properties. The interesting nanocomposite coatings formed by employing the PEO process is another highlight of the present summary. The main attributes of the PEO discharge process, such as the spectrum of plasma and the electric discharge characteristics, have been explained in the succeeding sections with few case studies.

Since the PEO process reforms the microstructure of the coating, it reduces the residual stresses and creates a more homogeneous coating overall. To gain a deeper insight into the coatings formed, the microstructure properties are elaborated. It is equally important to study the mechanical and tribological properties of the coating. The influence of environmental conditions such as corrosion, thermal, and optical properties affecting the PEO process is discussed. The concluding section discusses the various application of PEO coatings along with some of the latest technologies that can be combined with the PEO process. Finally, the authors believe that there can be more studies carried out on modifying the process as well as the coating. Interdisciplinary fields will play important roles in expanding the research related to this field. There is much room for research in PEO due to the customizability of the process.

## Figures and Tables

**Table 1:** Major differences between PEO & hard anodization (HA) process. Adapted from [12,16].

Surface Treatment Parameters	Differences
Voltage and electric current density	Higher for PEO than HA
Coating deposition rate	PEO is faster than HA
Oxidation process mechanism	PEO includes plasma reactions on top of normal HA electrochemical reactions.
Microstructure	PEO has both crystalline and amorphous porous outer layers, whereas HA has only an amorphous porous layer.
Oxidation and corrosion resistance	Higher for PEO than HA
Hardness	PEO is harder than HA
Resistance to wear	PEO resists wear better than HA
Thermal protection	Relatively higher for PEO than HA
Electrolyte	Alkaline in nature for PEO and acidic in nature for HA
Dielectric strength	Higher in value for PEO and lower for HA

**Table 2:** Composition and concentration of electrolytes employed. Adapted from [101].

Electrolyte	Composition	Concentration (M)
1	NaH_2_PO_4_ Ca(CH_3_COO)_2_	0.030 0.020
2	NaH_2_PO_4_ Ca(CH_3_COO)_2_ NA_2_(EDTA)	0.020 0.013 0.120
3	Ca(CH_3_COO)_2_ Na-Beta G	0.055 0.012
4	Ca(H_2_PO_4_)_2_ HMP NA_2_(EDTA) Ca(CH3COO)_2_	0.020 0.016 0.030 0.028
5	Ca(H_2_PO_4_)_2_ NA_2_(EDTA) Ca(CH_3_COO)_2_	0.050 0.045 0.036

**Table 3:** List of some commonly used additives in the PEO process.

Additive Particles	Substrate Material	Electrolyte	Properties	Reference
Polytetrafluoroethylene (PTFE)	(a) Al 2024 (b) AZ91D	Na_2_SiO_3_ + KOH Na_2_SiO_3_ + Na_2_B_4_O_7_ + KOH	Reduces coefficient of friction, provides chemical inertness	[113,114]
Silver (Ag)	(a) Ti6Al4V (b) Al_2_O_3_	C_4_H_8_CaO_5_ + Na_3_PO_4_ Na_3_C_6_H_5_O_7_ + C_6_H_8_O_7_ + Na_2_SiO_3_	Provides anti-bacterial property for biomedical application	[115,116]
MoS_2_	AZ31	K_3_PO_4_ + NaAlO_2_	Reduces coefficient of friction, provides high hardness	[117]
Clay	AM50	Na_3_PO_4_ + KOH	increases the density of the coating at low currents	[3]
ZrO_2_	AZ91	Na_2_SiO_3_ + NaOH + Na_2_ SiF_6_ + K_2_ZrF_6_ + ZrO_2_	Provides superior mechanical and electrochemical properties	[118]
SiO_2_	AM50	KOH + Na_3_PO_4_	Provides superior wear resistance	[57]
TiO_2_	Mg alloy	KOH + NaAlO_2_	Improves formation of a compact coating, improves corrosion resistance	[119]
Si_3_N_4_	AZ31	K_3_PO_4_ + NaAlO_2_	Provides high hardness, corrosion resistance, and good adhesion	[120]
Al_2_O_3_	AZ31B	NaOH + Na_2_SiO_3_	Provides improved corrosion resistance and hardness	[121]
SiC	AZ31	NaAlO_2_ + Na_2_ SiO_3_ + KOH (aluminate-silicate) Na_3_PO_4_ + KOH (phosphate)	Provides better wear and corrosion resistance, produces optimum hardness	[122]
Graphite	EV31A	Na_5_P_3_O_10_ + Na_2_SiO_3_ + NaOH	Reduces coefficient of friction, provides higher thickness and harness	[123]
Calcium acetate	AZ31	Na_3_PO_4_·12H_2_O + KOH	Provides stronger adhesion, increase in thickness, and improved corrosion resistance	[124]
FeSO_4_	1010AA	Na_2_SiO_3_ + KOH + Si_3_N_4_	Provides improved wear and corrosion resistance	[125]
Graphene oxide	AZ31	Na_3_PO_4_·12H_2_ O + KOH Na_2_HPO_4_ + NAF + Na_3_C_6_ H_5_O_7_ +NaC_12_ H_25_SO_4_	Provides optimum corrosion resistance and a uniform surface	[126]
Glycerol	AM50	Na_2_SiO_3_ + KOH	Provides better corrosion resistance	[127]
Phosphate	Mg-8.5 Li	Na_2_SiO_3_ + KOH + KF	Provides higher thickness, hardness, and wettability	[128]
Carbon nano tubes (CNT)	AZ31	KOH + KF + Na_2_SiO_3_	Provides improved corrosion resistance	[129]
ZnO	AZ91	K_3_PO_4_·3H_2_O + KOH	Provides improved corrosion resistance and enhanced bioactivity	[130]

**Table 4:** Table highlighting applications of few alloys subjected to PEO process.

Metal/Alloy	Application	Reference
Ti_6_Al_4_V	Biomedical	[205]
Ti_6_Al_7_Nb	Biomedical	[206]
Ti_48_Al_2_Cr_2_Nb	Aerospace, biomedical	[207]
Ti_13_Nb_13_Zr	Biomedical	[208]
NiTi	Biomedical	[209]
Tantalum (Ta)	Biomedical	[210]
Mg-Ca	Biomedical	[211]
AZ31	Biomedical, aerospace	[163,212]
AZ80	Automobile, electronics	[213]
AZ91	Automobile, aerospace, electronics	[185,214]
AM50	Aerospace, automobile	[163,215]
AM60	Aerospace, automobile	[216]
ACM522	Automobile	[217]
2024 Al	Aerospace, automobile	[218]
7075 Al	Marine	[219]
6061 Al	Aerospace	[155,202]
6082 Al	Automobile	[220]
356 Al	Automobile	[221]
Niobium (Nb)	Biomedical	[160]

## Data Availability

Not applicable.

## Abbreviations

PEO	Plasma Electrolytic Oxidation
PVD	Physical vapor deposition
CVD	Chemical vapor deposition
AC	Alternating current
DC	Direct current
OEM	Optical emission spectroscopy
HA	Hydroxyapatite
SEM	Scanning electron microscope
FESEM	Field emission scanning electron microscope
KOH	Potassium hydroxide
K_4_P_2_O_7_	Potassium pyrophosphate
K_3_PO_4_	Potassium triphosphate
SBF	Simulated body fluid
XRD	X-ray diffraction
EDTA	Ethylene diamine tetra acetic acid
PTFE	Poly tetra fluoro ethylene
MoS_2_	Molybdenum disulfide
ZrO_2_	Zirconium dioxide
SiO_2_	Silicon dioxide
TiO_2_	Titanium dioxide
Si_3_N_4_	Silicon nitride
SiC	Silicon carbide
CNT	Carbon nano tube
ZnO	Zinc oxide
KF	Potassium fluoride
K_2_ZrF_6_	Potassium hexafluorozirconate
K_3_PO_4_·3H_2_O	Potassium phosphate trihydrate
Ca_3_(PO_4_)_2_	Calcium phosphate
CeO_2_	Ceria
LST	Laser surface texture
TC	Tungsten carbide
R_a_	Average surface roughness
COF	Coefficient of friction
RFMS	Radio frequency magnetron sputtering
CaP	Calcium phosphate
SP	Shot peening
AM	Additive manufacturing
